# Mast Cell Cytonemes as a Defense Mechanism against Coxiella burnetii

**DOI:** 10.1128/mBio.02669-18

**Published:** 2019-04-16

**Authors:** Soraya Mezouar, Joana Vitte, Laurent Gorvel, Amira Ben Amara, Benoit Desnues, Jean-Louis Mege

**Affiliations:** a Aix-Marseille Université, IRD, APHM, MEPHI, IHU-Méditerranée Infection, Marseille, France; b APHM, IHU-Méditerranée Infection, UF Immunologie, Marseille, France; c CRCM, CNRS UMR7258, INSERM U1068, Institut Paoli-Calmettes, Aix-Marseille Université, UM105, Marseille, France; University of Pittsburgh

**Keywords:** CD36, *Coxiella burnetii*, mast cells, TLR4, cytonemes

## Abstract

Mast cells (MCs) are critical mediators of inflammation; however, their microbicidal activity against invading pathogens remains largely unknown. Here, we describe a nonpreviously reported antibacterial mechanism used by MCs against Coxiella burnetii, the agent of Q fever. We show that C. burnetii interaction with MCs does not result in bacterial uptake but rather induces the formation of extracellular actin filaments named cytonemes. MC cytonemes express cathelicidin and neutrophil elastase and mediate the capture and destruction of entrapped bacteria. We provide evidence that MC cytoneme formation and microbicidal activity are dependent on the cooperation of the scavenger receptor CD36 and Toll-like receptor 4. Taken together, our results suggest that MCs use an extracellular sophisticated mechanism of defense to eliminate intracellular pathogens, such as C. burnetii, before their entry into host cells.

## Abstract (precis)

Mast cells (MCs) are found in tissues that are in close contact with external environment, such as skin, lungs, or intestinal mucosa but also in the placenta during pregnancy. If their role in mediating allergic conditions is established, several studies now highlight their importance during infection with extracellular pathogens. This study showed a new and effective antimicrobial mechanism of MCs against Coxiella burnetii, an intracellular bacterium whose infection during pregnancy is associated with abortion, preterm labor, and stillbirth. The data reveal that in response to C. burnetii, MCs release extracellular actin filaments that contain antimicrobial agents and are capable to trap and kill bacteria. We show that this mechanism is dependent on the cooperation of two membrane receptors, CD36 and Toll-like receptor 4, and may occur in the placenta during pregnancy by using *ex vivo* placental MCs. Overall, this study reports an unexpected role for MCs during infection with intracellular bacteria and suggests that MC response to C. burnetii infection is a protective defense mechanism during pregnancy.

## INTRODUCTION

Mast cells (MCs) are hematopoietic cells residing in tissues that occupy a strategic position at host-environment interfaces, such as skin and mucosae. They are characterized by a high content of electron-dense secretory granules containing high-performance mediators, such as histamine, amines, serotonin, and proteases, such as tryptase and chymase, and express CD117 (c-kit) and IgE (FcεR1) receptors (1). They are well-known as immune effectors of anaphylaxis, but their role in defending against pathogens is emerging. MCs contribute to antibacterial immunity via multiple mechanisms. They are equipped with microbial sensors, such as Toll-like receptors (TLRs), including TLR2 and TLR4, and are involved in the recognition of Gram-positive and -negative bacteria (2–4). In response to lipopolysaccharide (LPS) or bacterial pathogens, MCs release inflammatory cytokines that mediate the recruitment of immune cells and secrete antimicrobial agents, including cathelicidin (LL-37) and neutrophil elastase (5). MCs are able to ingest and kill extracellular bacteria, such as Staphylococcus aureus, through an endosome-lysosome pathway (6) or to use an extracellular antimicrobial mechanism consisting of the release of extracellular traps (ETs) (7). Similarly to those formed by neutrophils, these ETs are composed of antimicrobial peptide-lined DNA projections, allowing the rapid immobilization and killing of microorganisms (7).

Coxiella burnetii is an intracellular bacterium responsible for Q fever, an acute infectious disease that may become persistent in specific clinical contexts (8). In myeloid cells, such as monocytes/macrophages and dendritic cells, C. burnetii survives and replicates, but it has nevertheless been shown to infect other cell types, including trophoblasts and adipocytes (9–12). C. burnetii is recognized by myeloid cells through αvβ3 integrin and TLR2/TLR4-dependent mechanism, leading to cytokine production and cytoskeleton reorganization (13, 14). C. burnetii is also able to subvert immune responses by interfering with uptake mechanisms and phagosome biogenesis (15), stimulating the production of interleukin-10 (IL-10), and promoting the expansion of regulatory T cells (13).

In the present study, we report that MCs were microbicidal for the intracellular pathogen C. burnetii through a nonpreviously reported mechanism. Indeed, MCs have released cytonemes consisting of long extensions of F-actin enriched with cathelicidin and neutrophil elastase. These structures trapped and killed C. burnetii organisms. Cytoneme-mediated killing of C. burnetii was under the control of the cross talk between TLR4 and CD36. These results suggest that MCs have shaped an extracellular sophisticated mechanism of defense to eliminate intracellular pathogens before their entry in immune cells.

## RESULTS

### MCs kill C. burnetii through an extracellular mechanism.

**FIG 1:**
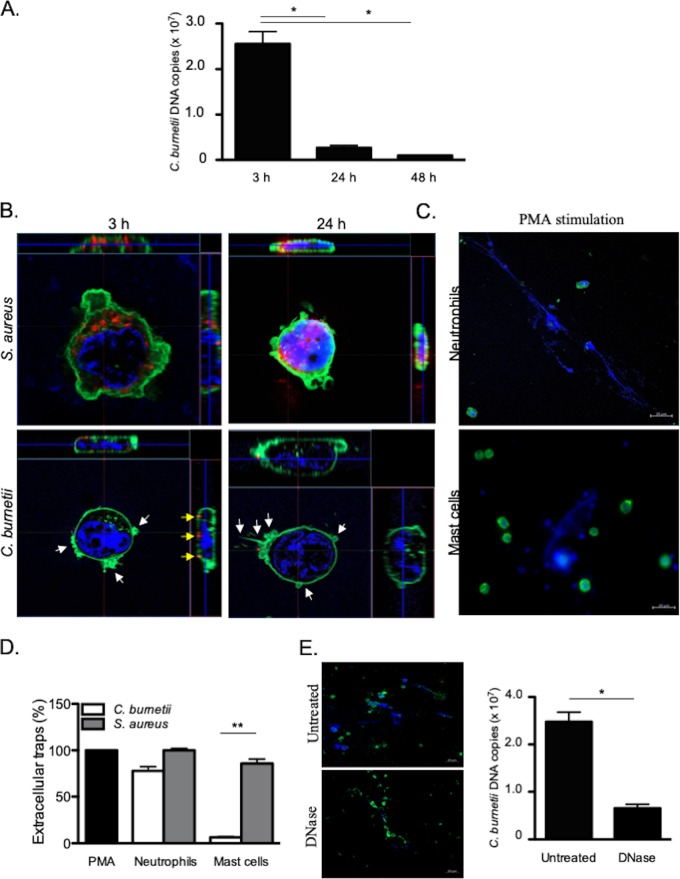
Extracellular killing of C. burnetii. HMC-1.2 cells (1 × 10^6^) were incubated with C. burnetii (50 bacteria per cell) for different periods of time. (A) After the cells were washed, the number of C. burnetii DNA copies was determined by qPCR. (B) Confocal sections of MCs incubated with S. aureus (top panel) or C. burnetii (bottom panel). Bacteria (red), F-actin (green), nucleus (blue), and cytoskeletal reorganization (white arrows) are indicated. Bacteria located on the MC membrane are represented by yellow arrows. (C and D) The extracellular traps released by neutrophils or MCs incubated with PMA, C. burnetii, or S. aureus for 3 h were observed (DNA in blue and F-actin in green) (C) and quantified by evaluating the release of fluorescent DNA (D). The results, expressed as relative to PMA-stimulated cells, are the means plus standard deviations (SD) (error bars) for triplicate samples from at least three independent experiments. **, *P ≤ *0.01. (E) The number of C. burnetii DNA copies was determined by qPCR in MCs incubated with bacteria for 3 h and then treated with 50 U/ml DNase for 10 min to eliminate ETs. The results are expressed as the means plus SD for triplicate samples from at least three independent experiments. *, *P ≤ *0.05.

HMC-1.2 cells were incubated with C. burnetii (50 bacteria per cell) for different periods of time, and the number of bacterial DNA copies was determined by qPCR. After 3 h of infection, more than 10 (7) C. burnetii DNA copies were detected. This number markedly decreased by 90% after 24 and 48 h (Fig. 1A). We questioned whether the decrease in the number of bacterial DNA copies reflected the uptake and elimination of bacteria by MCs. We assessed the uptake of C. burnetii by confocal microscopy, using S. aureus as the control. S. aureus organisms were found within MCs at 3 h postinfection (p.i.), and the bacterial burden increased at 24 h p.i. (Fig. 1B). In contrast, no C. burnetii organisms were found within MCs at 3 and 24 h p.i, as opposed to monocytes which are permissive cells for C. burnetii (see Fig. S1 in the supplemental material) as we previously described (16). It is noteworthy that some C. burnetii organisms were observed at the surfaces of MCs with intense F-actin rearrangements (Fig. 1B). The decreased number of C. burnetii DNA copies and the defective uptake of bacteria suggested that an extracellular antimicrobial mechanism was employed by MCs to eliminate C. burnetii. Since ETs are used by neutrophils and MCs to eliminate different types of bacteria (7), we quantified the release of ETs in response to C. burnetii and S. aureus and used PMA as a positive control. In neutrophils, C. burnetii and S. aureus induced a release of ETs similar to that induced by PMA (Fig. 1C). In MCs, S. aureus triggered intense formation of ETs as in neutrophils, whereas C. burnetii induced the release of few ETs (Fig. 1D). Therefore, we wondered whether these rare traps were sufficient to eliminate C. burnetii organisms. For that purpose, we treated C. burnetii-incubated MCs with DNase, which is known to disrupt ETs (17). This treatment did not increase the number of intracellular C. burnetii DNA copies, suggesting that another extracellular mechanism is involved in the trapping and elimination of C. burnetii by MCs (Fig. 1E). Taken together, these results suggested that MCs use an ET-independent extracellular mechanism to kill C. burnetii.

10.1128/mBio.02669-18.1 FIG S1 Monocytes as permissive cells for C. burnetii. Confocal micrographs of monocytes incubated with C. burnetii for 4 h (MOI of 50:1). Bacteria (red), F-actin (green), and nucleus (blue) are indicated. Download FIG S1, PDF file, 1.9 MB. Copyright © 2019 Mezouar et al. 2019 Mezouar et al. This content is distributed under the terms of the Creative Commons Attribution 4.0 International license.

### C. burnetii induces the formation of cytonemes by MCs.

**FIG 2:**
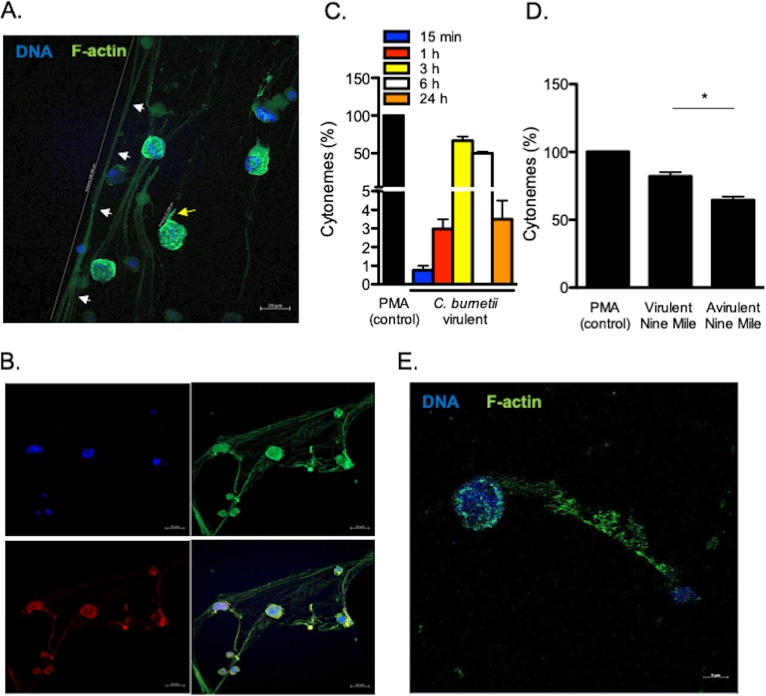
C. burnetii-induced formation of cytonemes. (A) Cytonemes (white arrows, distance 208.4 µm) and pseudopods (yellow arrow, distance 11.1 µm) stained with indicated markers were observed by confocal microscopy in MCs stimulated for 3 h. (B) Tubulin (red), F-actin (green), and DNA (blue) staining were evaluated on cytonemes. (C) The formation of cytonemes by MCs was quantified after stimulation with C. burnetii and expressed as a percentage relative to PMA as the positive control. (D) The formation of cytonemes was quantified after a 3-h incubation of MCs with virulent (Nine Mile) and avirulent (Nine Mile variant) bacteria. The results are expressed as a percentage relative to PMA stimulation. (E) The release of cytonemes by pMCs stimulated by C. burnetii for 3 h was observed by confocal microscopy, stained with the indicated markers. The results are expressed as the means plus SD for triplicate samples from four independent experiments. *, *P ≤ *0.05.

As C. burnetii markedly remodeled MC cytoskeleton, we analyzed the F-actin rearrangements induced by bacteria. We observed the release of extracellular thread-like membrane actin filaments (Fig. 2A). These structures were predominantly linear and distinct from pseudopods. They measured up to 200 µm in length and were composed of F-actin and tubulin (Fig. 2B), suggesting that they were similar to the cytonemes produced by MCs as previously described (18). The cytonemes appeared 15 min after C. burnetii stimulation; the number of cytonemes reached a maximum between 3 and 6 h (≥50%) and decreased thereafter (Fig. 2C). Several strains of C. burnetii have been described: they include the Nine Mile strain (the reference strain) and Guyana strain (the most virulent). We previously observed intense cell projections from monocytes stimulated by virulent C. burnetii compared to the avirulent variant (19). We wondered whether cytoneme formation was related to the virulence of C. burnetii. Avirulent variants of the Nine Mile strain poorly induced the formation of cytonemes compared to the virulent Nine Mile strain (Fig. 2D). Interestingly, the Guyana strain induced a significant increase of cytonemes formation compared to the PMA control (Fig. S2).

10.1128/mBio.02669-18.2 FIG S2 Small interference RNA of CD36 in HMC-1.2 cells. HMC-1.2 cells were transfected with siRNA directed against CD36 or the positive control (GAPDH) for 48 or 90 h. The quantification of CD36 mRNA expression in transfected HMC-1.2 cells was realized by qRT-PCR and was normalized to the value for the positive control (GAPDH). Download FIG S2, PDF file, 0.03 MB. Copyright © 2019 Mezouar et al. 2019 Mezouar et al. This content is distributed under the terms of the Creative Commons Attribution 4.0 International license.

As the formation of cytonemes was observed with a MC line, we wondered whether primary MCs responded similarly to C. burnetii. Therefore, we purified MCs from placenta, a tissue for which C. burnetii has a strong tropism (20). The placental MCs (pMCs) were identified by flow cytometry using FcεR1^+^/CD117^+^ (Fig. S3A) and tryptase staining (Fig. S3B). These pMCs were characterized by an ovoid nucleus, an irregular membrane, a metachromatic staining of granules, and the presence of several tryptase-positive cytoplasmic granules using MGG and toluidine blue (Fig. S3C), electron microscopy (Fig. S3D) and immunofluorescence (Fig. S3E). As observed for the MC cell line, C. burnetii also induced the formation of cytonemes in primary MCs (Fig. 2E). Altogether, these findings show that C. burnetii induces the formation of cytonemes by MCs and suggest that this phenotype depends, at least in part, on the virulence of the bacteria.

10.1128/mBio.02669-18.3 FIG S3 Isolation and characterization of pMCs. Placental cells from healthy donors were collected after enzymatic trypsinic digestion and Percoll cushion procedure. (A) The presence of pMCs in total placental cells was observed by flow cytometry using IgE^+^/CD117^+^ (A) and tryptase (B) staining. (C to E) Placental MCs were isolated using double magnetic bead selection, and their morphology and granularity were observed by colorations, including MGG (top panel) and toluidine blue (bottom panel, black arrows) (C), scanning electron microscopy (D), and confocal microscopy (E) (DNA [blue], F-actin [green], and tryptase [red]). Data are the mean ± SD for triplicate samples and are representative of three experiments. *, *P ≤ *0.05; **, *P ≤ *0.01. Download FIG S3, PDF file, 1.3 MB. Copyright © 2019 Mezouar et al. 2019 Mezouar et al. This content is distributed under the terms of the Creative Commons Attribution 4.0 International license.

### MC cytonemes capture and kill bacteria.

**FIG 3:**
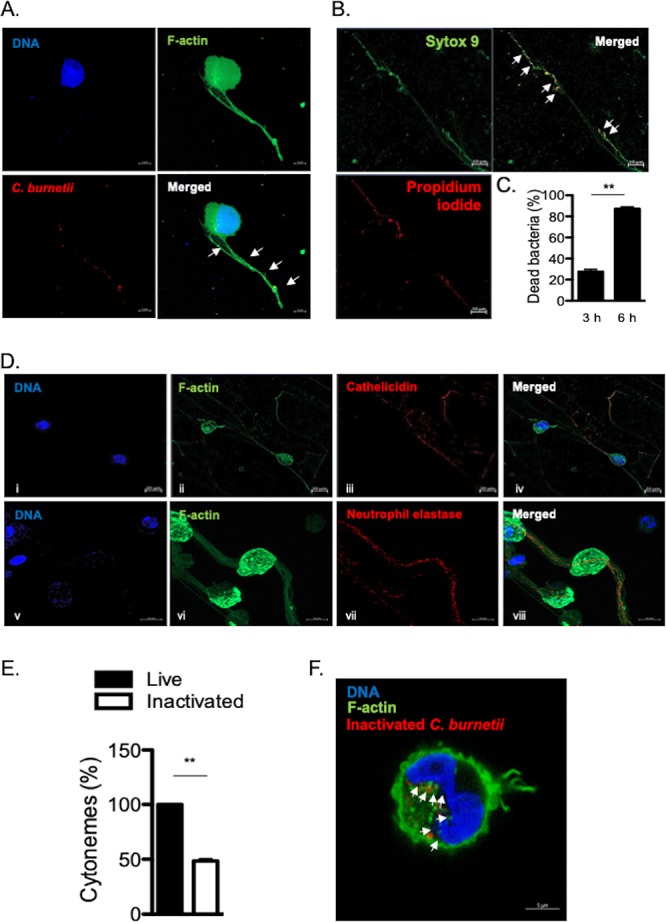
Cytonemes trap and kill C. burnetii. HMC-1.2 cells were incubated with C. burnetii (Nine Mile strain) for 3 h. (A) Bacteria (white arrows), colocalizing with cytonemes, appeared in yellow. (B) Bacteria entrapped in cytonemes were indicated with white arrows. Their viability was studied by immunofluorescence microscopy: Live bacteria were stained with Sytox 9 and appeared in green, whereas dead bacteria were stained with propidium staining and appeared in red. (C) The viability of bacteria entrapped in cytonemes was quantified at 3 and 6 h p.i. (D) Cathelicidin and neutrophil elastase were stained with specific Abs and were observed in red on MC cytonemes infected by C. burnetii with F-actin labeled in green and DNA in blue. (E) The percentage of cytonemes induced by heat-inactivated bacteria is expressed relative to the number of cytonemes induced by living bacteria. (F) The heat-inactivated bacteria were labeled in red within MCs (white arrows). F-actin and DNA are shown in green and blue, respectively. Data are the means plus SD for triplicate samples from three independent experiments. **, *P ≤ *0.01.

In order to understand the role of cytonemes in the MC response to C. burnetii, we incubated MCs with bacteria. We found that organisms were entrapped in cytonemes (Fig. 3A). After 3 h of contact between MCs and bacteria, approximately 20% of C. burnetii organisms were already dead and this number reached 80% after 6 h with the use of propidium iodide staining (Fig. 3B and C), suggesting that cytonemes were involved in C. burnetii killing. As MCs are known to secrete several antimicrobial products, we investigated their presence in cytonemes. MCs were incubated with C. burnetii for 3 h, and the distribution of antimicrobial agents, such as cathelicidin or neutrophil elastase, and extracellular F-actin, was studied by confocal microscopy. We found that both cathelicidin and neutrophil elastase colocalized with cytonemes (Fig. 3D), demonstrating that cytonemes were armed to kill entrapped bacteria.

Second, we wondered whether cytonemes may protect MCs by avoiding the internalization of virulent organisms. When MCs were incubated with heat-inactivated C. burnetii instead of living bacteria, cytoneme formation was significantly (*P* = 0.0042) reduced (Fig. 3E). Interestingly, we found that heat-inactivated bacteria were found inside MCs (Fig. 3F), suggesting that cytoneme formation was associated with restricted uptake of bacterial pathogens. Taken together, these results highlight the role of cytonemes in killing virulent C. burnetii and represent another way other than phagocytosis in MCs.

### Specific transcriptomic signature of C. burnetii-stimulated MCs.

**FIG 4:**
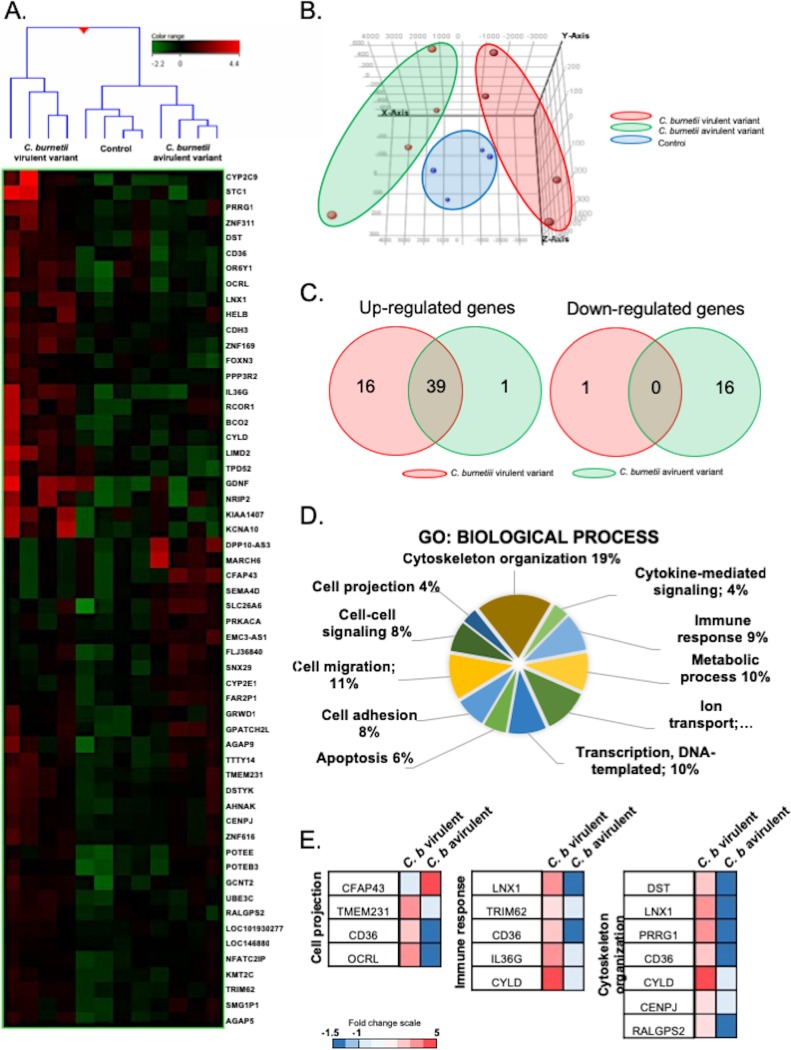
Transcriptional signature of MCs in response to C. burnetii. MCs were stimulated with C. burnetii (50 bacteria per cell) or left untreated for 8 h, and the total RNA was extracted prior to microarray analysis. (A) Up- and downregulated genes are indicated in red and green, respectively. (B) The relative distance between MCs stimulated with virulent C. burnetii (red), avirulent bacteria (green), or resting cells (blue) was assessed using principal-component analysis. (C) Venn diagrams showed the distribution of upregulated (left) and downregulated (right) genes in MCs stimulated with virulent C. burnetii (red) or avirulent variants (green). (D) Transcriptional analysis of modulated genes revealed several GO terms of biological process. (E) A modular analysis of the cell projection, immune response, and cytoskeletal organization of GO terms showed the genes involved and their modulation (up- and downregulation in red and blue, respectively).

To understand the molecular pathways involved in the formation of MC cytonemes, we studied the transcriptional signature of MCs stimulated with C. burnetii by whole-genome microarray. Hierarchical clustering revealed a specific pattern for MCs stimulated with virulent bacteria that induced the formation of cytonemes, whereas MCs stimulated with an avirulent variant clustered with unstimulated MCs (Fig. 4A). Principal-component analysis confirmed that the signatures of MCs, stimulated with virulent and avirulent bacteria, were clearly distinct (Fig. 4B). We found that 56 genes were differentially expressed in response to C. burnetii, and most of these genes were upregulated in response to virulent organisms, whereas downregulated genes were prominent in response to the avirulent variant (Fig. 4C). Genes involved in several biological processes were enriched using the Gene Ontology (GO) Consortium approach. They included genes involved in cytoskeleton organization, cytokine-mediated signaling, immune response, metabolic process, ion transport, transcription, apoptosis, cell adhesion, cell-cell signaling, and cell projection (Fig. 4D). Therefore, to validate our findings, we selected 10 genes (*TMEM231*, *OCRL*, *CYLD*, *IL36G*, *TRIM62*, *LNX1*, *DST*, *PRRG1*, *CENPJ*, and *RALGPS2*) for which we assessed the modulation by qRT-PCR (see Table S2 in the supplemental material).

### Interaction of CD36 with TLR4 in C. burnetii cell infection.

**FIG 5:**
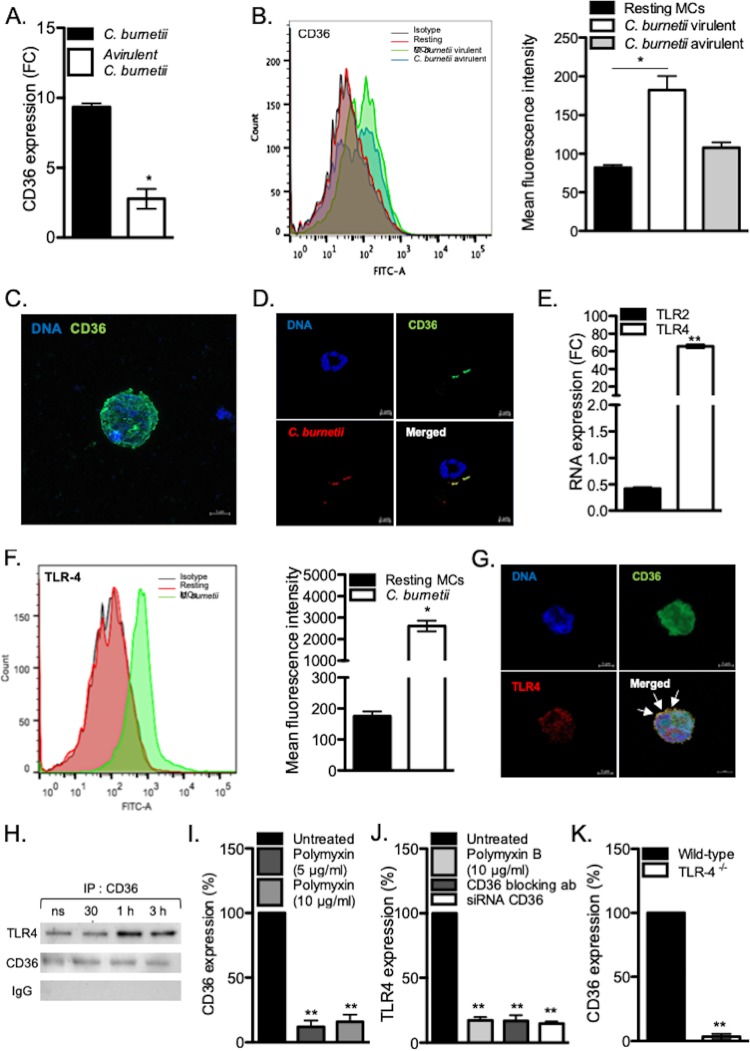
Involvement of CD36 and TLR4 in the response of MCs to C. burnetii. MCs were stimulated with C. burnetii or left untreated, and the modulation of the *CD36* and *TLR4* genes or encoded proteins was determined. (A) The expression of the gene encoding *CD36* was evaluated in MCs stimulated by C. burnetii for 8 h and normalized to the value for unstimulated cells. (B) The expression of CD36 protein was determined by flow cytometry with FITC-conjugated anti-CD36 on MCs stimulated with C. burnetii virulent and avirulent variants for 3 h. The results are expressed as mean fluorescence intensity. (C) Confocal microscopy revealed that the expression of CD36 (green) was essentially expressed at the membrane. (D) The staining of CD36 (green) and C. burnetii (red) showed that they colocalized (in yellow) at the MC membrane. (E) The expression of *TLR2* and *TLR4* transcripts was evaluated in MCs stimulated by C. burnetii for 8 h by qRT-PCR and normalized to the value for unstimulated cells. (F) The modulated expression of TLR4 protein in response to C. burnetii was determined by flow cytometry and expressed as mean fluorescence intensity. (G) Confocal microscopy showed that TLR4 (red) and CD36 (green) colocalized (yellow) in C. burnetii-stimulated MCs (DNA appeared in blue). (H) MCs were stimulated with C. burnetii for different periods. Immunoprecipitations were immunoblotted with TLR4, CD36, or irrelevant Abs. (I and J) The expression of the *CD36* (I) and *TLR4* (J) genes was evaluated by qRT-PCR in MCs stimulated with C. burnetii for 8 h and treated with CD36 blocking Abs or polymyxin B or after siRNACD36 transfection. The results are normalized to the values for untreated MCs. (K) The expression of *CD36* gene expression was assessed in TLR4^−/−^ BMdMCs and normalized to the value for wild-type BMdMCs. Data are the means plus SD for triplicate samples from four independent experiments. *, *P ≤ *0.05; **, *P ≤ *0.01.

Among the modulated genes, we found that the gene encoding CD36 was found in three GO terms, including cell projection, immune response, and cytoskeleton organization, and its expression was upregulated in response to virulent bacteria compared to the avirulent variant (Fig. 4E). The analysis of CD36 expression by qRT-PCR and flow cytometry confirmed microarray data and showed that CD36 expression was increased only in MCs stimulated with virulent bacteria (Fig. 5A and B). The confocal microscopy analysis revealed that CD36 was overexpressed as membrane clusters in MCs stimulated with C. burnetii (Fig. 5C). In addition, CD36 colocalized with C. burnetii at the surfaces of MCs (Fig. 5D). As CD36 is known to cooperate with TLRs to clear microbial infection (21) and TLR4 has been involved in actin remodeling in myeloid cells during C. burnetii infection (22), we investigated the expression of TLR2 and TLR4 in stimulated MCs. The expression of the *TLR4* gene, but not that of the *TLR2* gene, was dramatically upregulated in response to C. burnetii, as measured by qRT-PCR (Fig. 5E) and flow cytometry (Fig. 5F). In a second step, we wondered whether CD36 and TLR4 were associated in MCs stimulated by C. burnetii. Image overlay obtained by confocal microscopy showed that membrane CD36 and TLR4 colocalized at the surfaces of MCs incubated with C. burnetii (Fig. 5G). The direct interaction between CD36 and TLR4 was then assessed by immunoprecipitation experiment. We found that CD36 immunoprecipitated with TLR4 with a maximal intensity 1 h after incubation of MCs with C. burnetii (Fig. 5H). The cross talk between CD36 and TLR4 was further confirmed by inhibition experiments. First, inhibition of CD36 with blocking Abs (Fig. 5J) or transfection of MCs with a siRNA directed against *CD36* (Fig. S4), significantly reduced the expression of *TLR4* in stimulated MCs (Fig. 5I). In addition, polymyxin B, an inhibitor of LPS binding, reduced *TLR4* and *CD36* expression of C. burnetii-stimulated cells. Finally, in TLR4-deficient BMdMCs stimulated by C. burnetii, the expression of *CD36* was severely impaired compared to wild-type BMdMCs (Fig. 5K). These results indicated a direct cooperation between TLR4 and CD36 in the response of MCs to C. burnetii.

10.1128/mBio.02669-18.4 FIG S4 Low interference of CD36 RNA in HMC-1.2 cells. HMC-1.2 cells were transfected with siRNA directed against CD36 or the positive control (GAPDH) for 48 or 90 h. The quantification of CD36 mRNA expression in transfected HMC-1.2 cells was realized by qRT-PCR and was normalized to the value for the positive control (GAPDH). Download FIG S4, PDF file, 0.03 MB. Copyright © 2019 Mezouar et al. 2019 Mezouar et al. This content is distributed under the terms of the Creative Commons Attribution 4.0 International license.

### Role of CD36/TLR4 complex in cytoneme formation.

**FIG 6:**
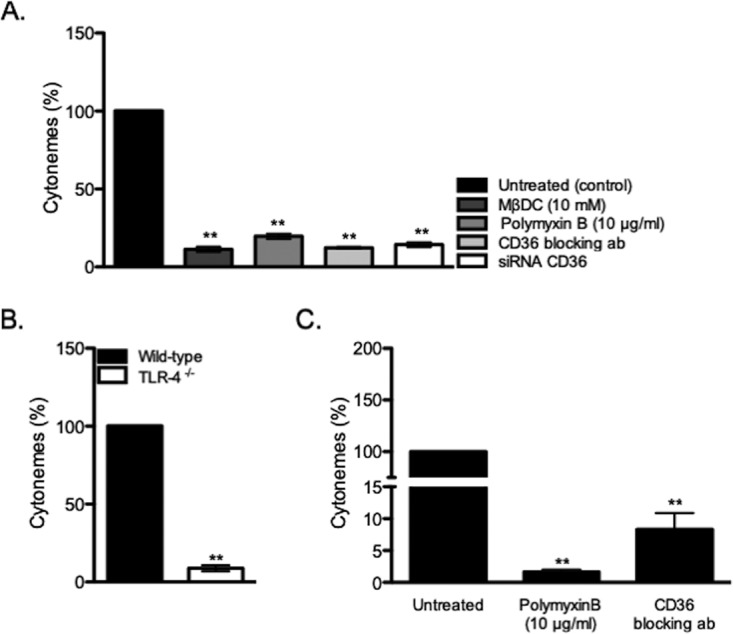
CD36/TLR4 cooperation. MCs were stimulated with C. burnetii for 3 h. (A) The percentage of cytonemes was calculated after treatment of stimulated MCs during 10 min with MβDC, polymyxin B, CD36 blocking Abs, or after siRNA CD36 transfection compared to untreated cells. (B) The percentage of cytonemes was calculated in TLR4-deficient BMdMCs compared to wild-type BMdMCs. (C) Placental MCs from healthy donors were collected after enzymatic digestion, Percoll cushion procedure, and magnetic selection. The release of cytonemes by pMCs stimulated by C. burnetii for 3 h were quantified after polymyxin B sulfate or CD36 blocking antibody treatments. Data are the means ± SD for triplicate samples and are representative of four independent experiments. **, *P ≤ *0.01.

Since the CD36/TLR4 cooperation is initiated by the interaction of C. burnetii with MCs, we wondered whether the CD36/TLR4 complex was involved in the formation of cytonemes. We therefore measured the cytoneme formation in response to C. burnetii in the presence of CD36 inhibitors. CD36 siRNA or CD36 blocking Abs inhibited cytoneme formation (Fig. 6A). Similarly, polymyxin B that inhibited CD36 expression (Fig. 5I) also prevented the formation of cytoneme. Finally, in TLR4-deficient BMdMCs stimulated by C. burnetii, an inhibition of 85% of cytoneme formation was observed (Fig. 6B). We then confirmed these results in primary MCs and showed that the treatment of pMCs with polymyxin B or CD36 blocking Abs inhibited cytoneme formation (Fig. 6C). Taken together, these results indicate that the release of cytoneme by MCs depends on cooperation between CD36 and TLR4 in response to the challenge of C. burnetii.

## DISCUSSION

We have demonstrated here an antibacterial mechanism of MCs based on cytonemes that had never been reported before. It is established that bacteria are killed by innate immune cells via numerous intra- and extracellular mechanisms. The former are based on phagocytosis and phagosomal maturation, whereas the latter involve the formation of ETs and the release of bactericidal compounds (23). Microorganisms, including intracellular bacteria, have developed various strategies to subvert innate immune responses. Indeed, C. burnetii successfully infects macrophages by controlling phagocytosis and phagosome biogenesis (15, 24). We observed that MCs were able to kill C. burnetii without internalizing it, suggesting an extracellular microbicidal mechanism. This result is markedly distinct from S. aureus, which is killed by MCs via internalization and ET formation (6). While C. burnetii induces the formation of ETs as efficiently as S. aureus in neutrophils, C. burnetii is a poor inducer of ETs in MCs, which appeared insufficient to kill bacteria.

We also provided evidence that MCs use cytonemes for a microbicidal effect toward C. burnetii. Cytonemes were initially associated with cell-cell communication (25, 26). Although these cytoskeletal structures may participate in host defense, their specific antimicrobial role has not been described thus far. Cytonemes have been implicated in cell-to-cell spreading of virions, such as human immunodeficiency virus type 1 and human T cell leukemia virus type 1 (HTLV-1) (27, 28, 29). Recently, Hashimoto et al. reported that macrophage cytonemes enable the rapid transfer of HTLV-1 to surrounding cells (30). In contrast, neutrophil cytonemes are involved in the tethering of bacteria, such as S. aureus, Salmonella enterica serovar Typhimurium, and Helicobacter pylori. This capture of bacteria by neutrophil cytonemes allows internalization and subsequent intracellular destruction (31). The microbicidal activity of neutrophil cytonemes is probably based on the release of bactericidal molecules, such as lactoferrin, lipocalin, myeloperoxidase, defensins, and cathepsin G (32). We reported here that cytonemes entrapped C. burnetii and reduced its viability. The latter response is likely mediated by elastase and cathelicidin, which colocalized with bacteria within cytonemes. We also discovered that cytoneme-mediated killing of C. burnetii is associated with the absence of phagocytosis, whereas cytonemes and phagocytosis are associated, during the interaction of S. aureus, with MCs. The dissociation of cytoneme formation from phagocytosis was related to bacterial viability, since inactivated bacteria were internalized and did not induce significant cytoneme formation. The prevention of phagocytosis in MCs is an active process reminiscent of what we reported in macrophages and monocytes infected with virulent C. burnetii (19, 33). Indeed, C. burnetii induced cytoskeletal reorganization of macrophages, such as F-actin protrusions, which was associated with phagocytosis prevention (10, 22). Manipulation of the cytoskeleton organization in macrophages restores phagocytosis, thus establishing a direct link between cytoskeleton modulation and phagocytosis interference (33, 34). We hypothesize that C. burnetii-induced cytonemes alert the immune system since it has been reported that externalized F-actin acts as a danger signal (35). Finally, in line with Manfredi et al. who proposed a choice between phagocytosis and generation of ETs for neutrophils during inflammation and infection (36), we think that MCs make a choice between MC formation and phagocytosis in the context of C. burnetii infection.

Our study also described that a functional cooperation between CD36 and TLRs is necessary for cytoneme formation. Indeed, we provide evidence that CD36 associates with TLR4, thus forming a molecular complex that is involved in the production of cytonemes and entrapping C. burnetii. This is consistent with previous reports in which CD36 mediated signal transduction for TLR4 (37, 38, 39). The role of CD36 in the immune response to microorganisms remains unclear. Some studies evoked a direct role of CD36 in inflammatory response or in pathogen recognition (40, 41). Indeed, Stuart et al. reported that CD36 may be a bacterial receptor for S. aureus, its cytoplasmic C-terminal extremity being involved in bacterial internalization (42). The transfection of CD36 into HeLa cells enhances the uptake of bacteria, including Escherichia coli, Klebsiella pneumoniae, *S.* Typhimurium, S. aureus, and *Enterococcus faecalis* (40). In addition, CD36 is involved in the internalization of LPS by endothelial or MCs (25, 43, 44). Future studies are required to address the precise mechanism underlying CD36/TLR4 cross talk during C. burnetii infection.

The formation of cytonemes in response to C. burnetii was not restricted to MC lines or murine MCs derived from bone marrow. It was also observed in primary MCs isolated from the human placenta. C. burnetii is known for its tropism for placenta tissue, and Q fever is a major risk for pregnancy and impaired fetal development (20). We previously described that C. burnetii interacts with dendritic cells from placenta and replicates in trophoblasts (11, 45). Here, we report that MCs from placenta present an extracellular mechanism to kill bacteria in the early phases of C. burnetii infection. The role of MCs in promoting host resistance in bacterial infection is documented (46–48). Thus, we can speculate that during C. burnetii infection, MCs play a role of protection at the placenta level by intercepting extracellular pathogens and limiting abortion. This finding may also explain why trophoblasts constitute a niche for C. burnetii (11). However, these hypotheses have to be clarified by careful examination of organism distribution in the naturally infected placenta in order to provide insights into the roles of individual cell types in abortion during Q fever.

In conclusion, our data described a new extracellular bactericidal mechanism based on the release of cytonemes by MCs. Cytonemes were involved in the capture of virulent C. burnetii and the destruction of entrapped bacteria mediated by antimicrobial peptides. We also showed that the formation of cytonemes requires the CD36/TLR4 complex. This report opens new perspectives in the antimicrobial activity of MCs and provides new insights into the role of cytonemes in the immune response.

## MATERIALS AND METHODS

### Cells.

The human mast cell line HMC-1.2 was generously provided by M. Arock (Paris, France) and cultured in Iscove’s modified Dulbecco’s medium (IMDM) (Life Technologies) supplemented with 10% fetal bovine serum (FBS), 100 IU/ml penicillin, and 50 µg/ml streptomycin (Life Technologies) at 37°C. In some experiments, placental MCs (pMCs) were isolated. Placenta from at-term healthy women were included after providing written informed consent and after approval was granted from the Comité d’Ethique d’Aix Marseille Université (number 08-012). Placenta tissue was digested with trypsin, and the cell suspension was deposited on a 25 to 60% Percoll cushion and centrifuged as previously described (49). Placental cells were collected, and pMCs were enriched by positive selection after FcεR1/CD117 staining (Miltenyi Biotec). In some experiments, bone marrow-derived MCs (BMdMCs) were differentiated as previously described (50). Briefly, wild-type mice were obtained from Charles River Laboratories, and TLR4-deficient mice were generously provided by L. Alexopoulou (Marseille, France). Bone marrow cells were flushed and incubated with IMDM containing 15% FBS, 10 ng/ml IL-3, and 10 ng/ml stem cell factor (Miltenyi Biotec). After 4 weeks of culture, the differentiation into BMdMCs was checked by flow cytometry using CD117 and FcεR1 as specific markers. About 98% of cultured cells were BMdMCs (data not shown). Human neutrophils and monocytes were isolated from blood samples from three healthy donors (Établissement Français du Sang, Marseille, France) using Percoll or Ficoll gradient, respectively, and incubated in RPMI 1640 (Life Technologies), as previously described (17).

### Bacteria.

Bacteria (Nine Mile and Guyana strains of C. burnetii) were prepared as previously reported (12). Avirulent variants of Nine Mile bacteria were obtained after repeated passages in L929 cells. Bacteria were stored at −80°C, their concentration was determined by Gimenez staining, and bacterial viability was assessed using the live/dead BacLight bacterial viability kit (Molecular Probes, Life Technologies) (51). Bacteria were inactivated at 95°C for 30 min. S. aureus (ATCC 25923) bacteria were grown on blood agar plates (bioMérieux) and quantified by flow cytometry (FACS BD Fortessa).

### Cell stimulation.

MCs and neutrophils (1 × 10^6^ cells/well) were incubated in 24-well plates pretreated with fibronectin (1 mg/well; Life Technologies) for 3 h. Adherent cells were stimulated with 100 µg/ml LPS, 25 nM phorbol-12-myristate-13-acetate (PMA) (MP Biomedicals), or bacteria (bacterium-to-cell ratio of 25 and 50 bacteria per cell for S. aureus and C. burnetii, respectively) at 37°C. The roles of CD36 and TLR4 were studied using MCs pretreated with 5 µg/ml CD36-blocking antibodies (Abs) (mouse IgG2a; Thermo Fisher Scientific) or 10 µg/ml of the TLR4 inhibitor polymyxin B sulfate (Sigma-Aldrich) for 10 min.

### Bacterial detection.

C. burnetii organisms were detected by quantitative PCR (qPCR) and immunofluorescence as previously described (12). The total DNA was extracted using the NucleoSpin kit (Macherey-Nagel). Quantitative PCR was performed using Sybr Green Technologies using a CFX (Bio-Rad) with 5 µl of DNA and specific primers targeting C. burnetii *com1* gene: sense (5′-GCACTATTTTTAGCCGGAACCTT-3′) and antisense (5′-TTGAGGAGAAAAACTGGATTGAGA-3′). C. burnetii organisms were also detected by immunofluorescence. In brief, MCs were fixed with 3% paraformaldehyde, permeabilized with 0.1% Triton X-100 for 5 min, and incubated with a 1/100 dilution of Q fever patient serum (11). After washing, MCs were incubated with Alexa Fluor 647-conjugated Abs. S. aureus was labeled with the fluorochrome DID (4-(4-(dihexadecylamino)styryl)-*N*-methylpyridinium iodide) (Thermo Fisher Scientific) for 20 min at 37°C.

### Cytonemes and extracellular traps.

The quantification of ET release was based on the evaluation of the area of labeled extracellular DNA filaments using an Axio Scan coupled with Hamamatsu sCOMS Flash 4 camera (Zeiss). The area of extracellular DNA of ETs was quantified on five different fields as previously described (52, 53). The results are expressed as a percentage relative to the PMA-stimulated cells as a positive control (cells stimulated with PMA) (54). Similarly, the evaluation of cytoneme formation over time was realized by assessment of area of extracellular F-actin filaments and expressed as a percentage relative to the PMA control, as depicted in Fig. S5 in the supplemental material. In some experiments, stimulated MCs were treated with 50 U/ml DNase (Sigma-Aldrich) for 30 min at the end of the experiment to disrupt ETs as previously described (17). The cytonemes were quantified using a similar method based on the release of extracellular F-actin labeled with phalloidin-488. To inhibit the formation of cytonemes, MCs were pretreated with 10 mM methyl-β-D-cyclodextrin (MβDC) (Sigma-Aldrich) for 10 min as previously described (18).

10.1128/mBio.02669-18.5 FIG S5 Cytoneme quantification. The area of extracellular F-actin filaments, referred as cytonemes, was quantified relative to PMA-stimulated MCs at 15, 60, and 180 min after stimulation. Download FIG S5, PDF file, 0.03 MB. Copyright © 2019 Mezouar et al. 2019 Mezouar et al. This content is distributed under the terms of the Creative Commons Attribution 4.0 International license.

### MC phenotyping and cytoneme staining.

MCs were incubated with Abs directed against CD36 (mouse IgG1; Beckman Coulter), CD117 (mouse IgG1; Beckman Coulter), FcεR1 (mouse IgG1; Bühlmann Laboratories), TLR4 (mouse IgG1; BD Pharmigen), tryptase (mouse IgG1; Thermo Fisher Scientific), neutrophil elastase (rabbit IgG; Abcam), cathelicidin (rabbit IgG; Thermo Fisher Scientific), tubulin (mouse IgG1; Thermo Fischer Scientific), or appropriate isotype controls for 1 h. After the cells were washed, secondary Abs conjugated to Alexa Fluor 647 goat anti-rabbit or anti-mouse IgG1 (Life Technologies) were added to MCs for 30 min. Stained cells were then analyzed by confocal microscopy using an LSM 800 Airyscan confocal microscope (Zeiss) or by flow cytometry (10,000 events/acquisition) using a FACS BD Fortessa flow cytometer (BD Biosciences). The results of flow cytometry are expressed in mean fluorescence intensity (MFI), as calculated by the FlowJo software vX.0.7.

### Microarray and data analysis.

MCs (1 × 10^6^ cells/well) were stimulated or not stimulated with C. burnetii for 8 h, and the total RNA was extracted using a RNeasy Mini kit followed by DNase treatment (Qiagen) to perform microarray experiment as described above (11). The 4X44K Human Whole Genome G4112F microarrays (Agilent Technologies), representing 45,000 probes, were used. Sample labeling and hybridization were performed using one-color microarray-based gene expression analysis. Four samples per experimental condition were included in the analysis. Slides were scanned with a pixel size of 5-µm resolution with a G2505C DNA microarray scanner (Agilent Technologies), and data were analyzed with Feature Extraction Software 10.5.1. The selected probes were filtered for differentially expressed genes using an absolute fold change (FC) of ≥1.5. The functional annotation was performed using DAVID Bioinformatics Resources (55, 56). The modulation of some genes was confirmed by quantitative reverse transcription-PCR (qRT-PCR) using the MMLV-RT kit (Life Technologies) and SYBR Green Fast Master Mix (Roche Diagnostics). Confirmation experiments were conducted using specific primers designed with Primer3 software (Table S1). The results were normalized using the housekeeping gene *actb* gene encoding β-actin and are expressed as the mean of FC = 2^−ΔΔCt^ in which ΔΔCt = (Ct_Target_ − Ct_Actin_)_assay_ − (Ct_Target_ − Ct_Actin_)_control_. The threshold cycle (Ct) was defined as the number of cycles required to detect the fluorescent signal. The expression of genes was considered modulated when the FC was ≥1.5.

10.1128/mBio.02669-18.6 TABLE S1 Sequences and gene names of specific primers used for qRT-PCR. Download Table S1, PDF file, 0.01 MB. Copyright © 2019 Mezouar et al. 2019 Mezouar et al. This content is distributed under the terms of the Creative Commons Attribution 4.0 International license.

10.1128/mBio.02669-18.7 TABLE S2 Microarray data validation by qRT-PCR. Fold change (FC) for microarray and qRT-PCR experiments of a sampling of modulated genes with GO terms identified by microarray analysis. The FC values were evaluated for MCs stimulated by virulent or avirulent C. burnetii for 8 h and normalized to the value for unstimulated cells by qRT-PCR. Data are the means for triplicate samples and are representative of two experiments. Download Table S2, PDF file, 0.03 MB. Copyright © 2019 Mezouar et al. 2019 Mezouar et al. This content is distributed under the terms of the Creative Commons Attribution 4.0 International license.

### Small interference RNA transfection (siRNA).

siRNAs directed against CD36 were purchased from Ambion (Life Technologies) and constructed with the following target sequences: sense (5′-CACUAUCAGUUGGAACAGAtt-3′) and antisense (5′-UCUGUUCCAACUGAUAGUaa-3′). HMC-1.2 cell line (1 × 10^6^ cells/well) were grown to 80% confluence and transfected with 5 nM CD36 siRNA for 6 h using Lipofectamine 2000 (Life Technologies), according to the manufacturer’s instructions (Life Technologies).

### Immunoprecipitation.

HMC-1.2 cells (1 × 10^7^ cells) were treated with C. burnetii (50 bacteria per cell) for the indicated periods, washed in ice-cold phosphate-buffered saline and lysed in 20 mM Tris-HCl (pH 7.4), 200 mM NaCl, 1 mM EDTA, 1% Triton X-100 with protease inhibitors as previously described (41). Protein lysate was incubated with 4 μg of anti-human CD36 (Thermo Fisher Scientific) or control IgG (mouse IgG2a; Beckman Coulter) overnight at 4°C and then incubated with protein G-Sepharose beads (Sigma-Aldrich) for 3 h at 4°C. Immunoblotting was performed on 10% polyacrylamide gels using anti-CD36 (rabbit IgG; Thermo Fisher Scientific) and anti-TLR4 (rabbit IgG; Thermo Fisher Scientific) Abs, and the signal were recorded using the ECL Plus reagent (Thermo Fisher Scientific).

### Statistical analysis.

Data were analyzed with GraphPad Prism 5.0c and Student’s *t* test. A *P* value of <0.05 was considered statistically significant.

### Accession number(s).

The data have been deposited in NCBI’s Gene Expression Omnibus (57) and are accessible though GEO series accession number GSE111971.
