# Supplementary material for: Mast Cell Cytonemes as a Defense Mechanism against Coxiella burnetii
Source: mBio. 2019 Apr 16;10(2):e02669-18. doi: 10.1128/mBio.02669-18 (PMC6469977; doi:10.1128/mBio.02669-18)

Figure S1. Monocyte as permissive cells for *C. burnetii*. Confocal pictures of monocytes incubated with *C. burnetii* for 4 hours (MOI 50:1). Bacteria are indicated in red, F-actin in green and nucleus in blue.

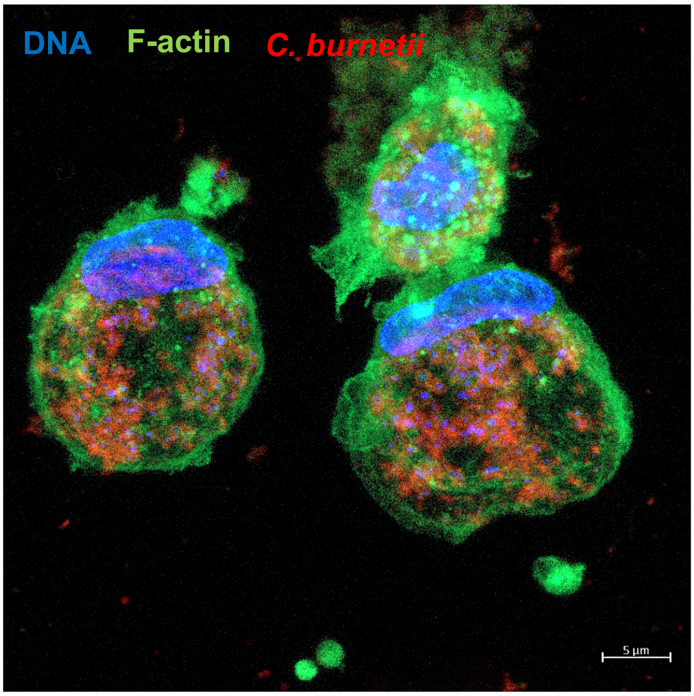

Supplement: FIG S1 [file mBio.02669-18-sf001.pdf]
