# Supplementary material for: Mast Cell Cytonemes as a Defense Mechanism against Coxiella burnetii
Source: mBio. 2019 Apr 16;10(2):e02669-18. doi: 10.1128/mBio.02669-18 (PMC6469977; doi:10.1128/mBio.02669-18)

Figure S2. MC cytonemes in response to Guyana strain

The formation of cytonemes was quantified after a 3 hours incubation of MCs with high virulent (Guyana) bacteria. The results are expressed in percentage relative to PMA stimulation.

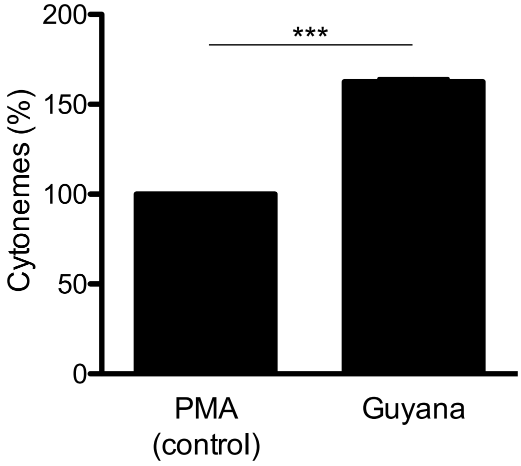

Supplement: FIG S2 [file mBio.02669-18-sf002.pdf]
