# Supplementary material for: Mast Cell Cytonemes as a Defense Mechanism against Coxiella burnetii
Source: mBio. 2019 Apr 16;10(2):e02669-18. doi: 10.1128/mBio.02669-18 (PMC6469977; doi:10.1128/mBio.02669-18)

Figure S3. Isolation and characterization of pMCs

Placental cells from healthy donors were collected after enzymatic trypsinic digestion and Percoll cushion procedure. **(A)** The presence of pMCs in total placental cells were observed by flow cytometry using FcεR1^{+}/CD117^{+} and **(B)** tryptase staining. **(C)** Placental MCs were isolated using double magnetic beads selection and their morphology and granularity were observed by colorations including MGG (upper panel) and toluidine blue (lower panel, black arrows), **(D)** scanning electron and **(E)** confocal microscopy (DNA (blue), F-actin (green) and tryptase (red)). Data are the mean ± SD of triplicate samples and are representative of three experiments. *$p \leq 0.05$ and **$p \leq 0.01$.

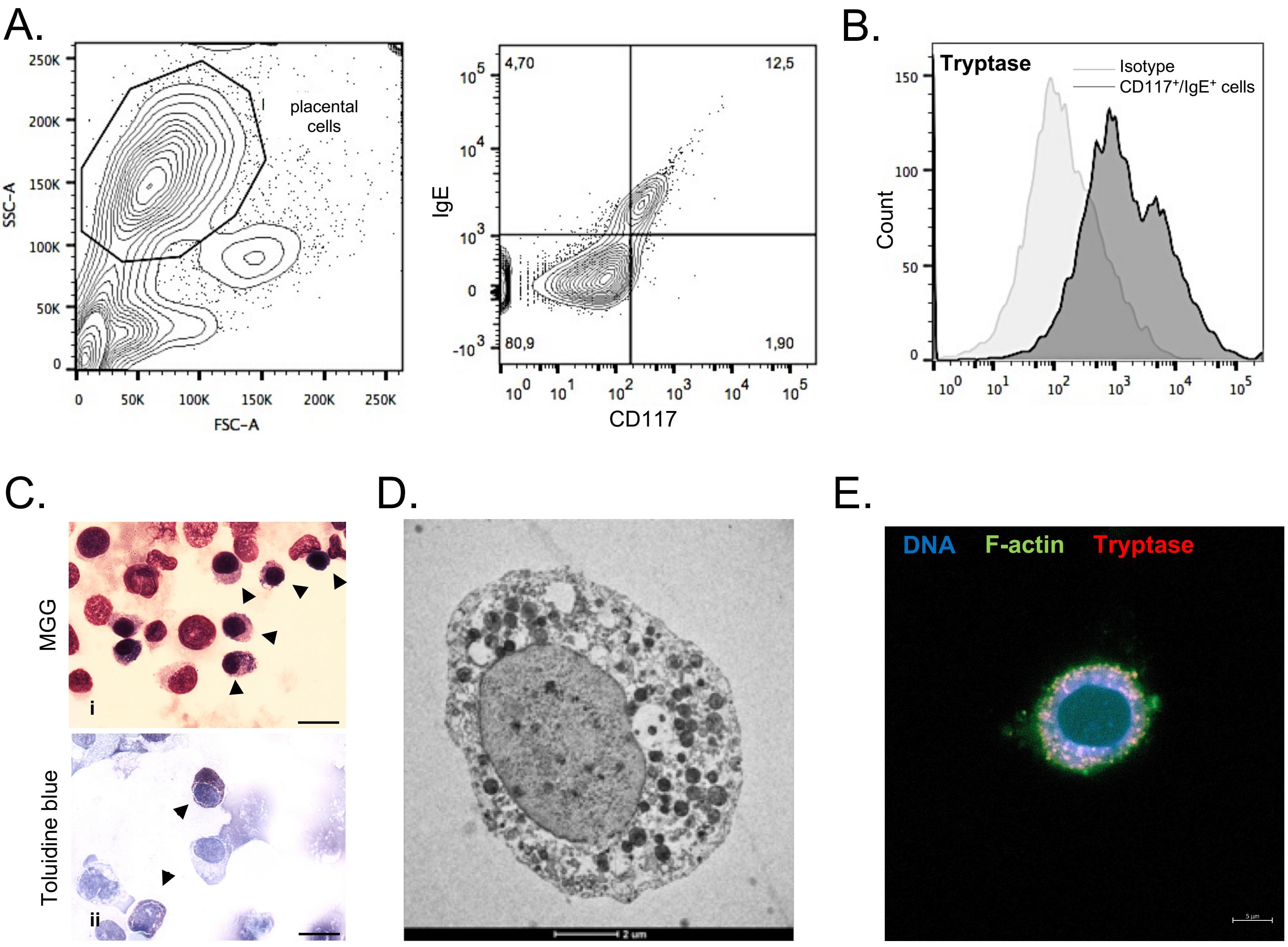

Supplement: FIG S3 [file mBio.02669-18-sf003.pdf]
