# Supplementary material for: Mast Cell Cytonemes as a Defense Mechanism against Coxiella burnetii
Source: mBio. 2019 Apr 16;10(2):e02669-18. doi: 10.1128/mBio.02669-18 (PMC6469977; doi:10.1128/mBio.02669-18)

Figure S4. Low interference of CD36 RNA in HMC-1.2 cells

HMC-1.2 cells were transfected with siRNA directed against CD36 or positive control (GAPDH) for 48 or 90 hours. The quantification of CD36 mRNA expression in transfected HMC-1.2 cells was realized by q-RTPCR and was normalized to positive control (GAPDH).

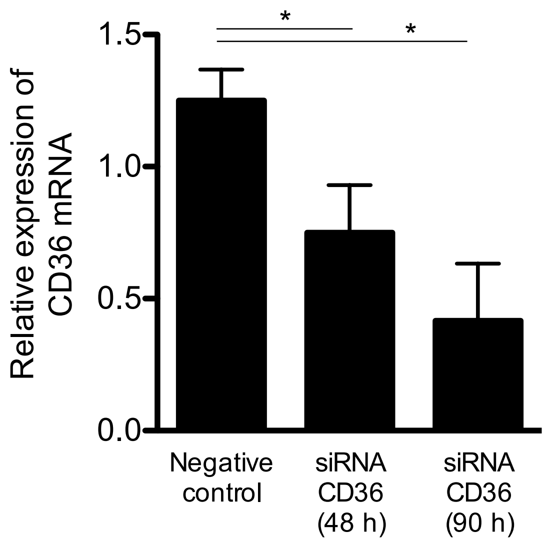

Supplement: FIG S4 [file mBio.02669-18-sf004.pdf]
