# Supplementary material for: Mast Cell Cytonemes as a Defense Mechanism against Coxiella burnetii
Source: mBio. 2019 Apr 16;10(2):e02669-18. doi: 10.1128/mBio.02669-18 (PMC6469977; doi:10.1128/mBio.02669-18)

Figure S5. Cytoneme quantification. The area of extracellular F-actin filament, as cytonemes, was quantified in PMA-stimulated MCs at 15, 60 and 180 minutes post-stimulation.

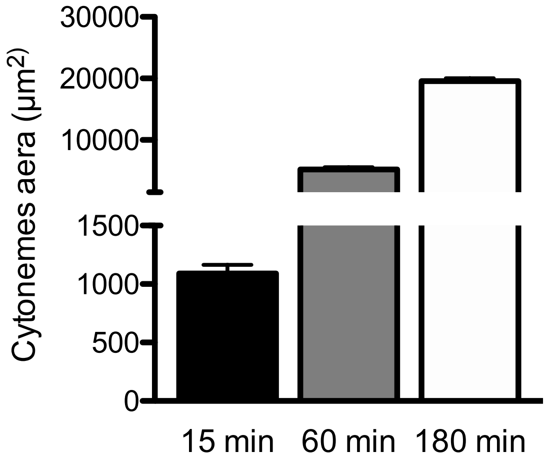

Supplement: FIG S5 [file mBio.02669-18-sf005.pdf]
