# Supplementary material for: Mast Cell Cytonemes as a Defense Mechanism against Coxiella burnetii
Source: mBio. 2019 Apr 16;10(2):e02669-18. doi: 10.1128/mBio.02669-18 (PMC6469977; doi:10.1128/mBio.02669-18)
Supplement: TABLE S1 [file mBio.02669-18-st001.pdf]

Table S1. Sequences and gene name of the specific primers used for qRT-PCR

Gene Symbol	Forward	Reverse
**TMEM231**	agaataccaagcagtccagtgg	gaagcagctccatgtgttcttg
**CD36**	ttgtaggctgcatcccatatct	tgtggtagtaacagggtacgga
**OCRL**	gccgtatgaaaagaccaatccg	tagaagccagatctccttggga
**CYLD**	ctcaagtccaccttcatccgaa	tcaaagaagagccaggcagaat
**IL-36G**	catgcaagtatccagaggctct	aatgatgggctggtctctcttg
**TRIM62**	tggaggttgcattccctacttc	cataagagggtgctgtgtgtct
**LNX1**	agaatgaccgtgtgttagccat	gagagattcaccggggtctttt
**DST**	ccacacccagcctaatgtaact	ttgtaactgacgaccctgagtg
**PRRG1**	ctaggctcggcagaaataggag	aaccctccccatgttttcttgt
**CENPJ**	gccacttgaaccactgaacttc	tcttcccatctgcactcacttc
**RALGPS2**	attagcttaaactggggccctc	cacccccttgtttgcagaagatg
