# Supplementary material for: Mast Cell Cytonemes as a Defense Mechanism against Coxiella burnetii
Source: mBio. 2019 Apr 16;10(2):e02669-18. doi: 10.1128/mBio.02669-18 (PMC6469977; doi:10.1128/mBio.02669-18)
Supplement: TABLE S2 [file mBio.02669-18-st002.pdf]

Table S2. Microarray data validation by qRT-PCR

Gene symbol	FC (virulent-C. burnetii)		FC (avirulent variant)	
	Microarray	qRT-PCR	Microarray	qRT-PCR
**TMEM231**	2.04	1.58	1.47	0.29
**OCRL**	2.06	1.65	-1.01	0.16
**CYLD**	2.88	1.89	1.02	1.12
**IL36G**	4.56	3.27	1.18	2.65
**TRIM62**	1.69	2.61	1.22	1.26
**LNX1**	2.27	1.45	-1.17	1.09
**DST**	1.76	1.62	-1.21	0.36
**PRRG1**	2.44	1.08	-1.03	0.78
**CENPJ**	1.54	1.23	1.19	1.58
**RALGPS2**	1.50	0.97	-1.02	1.43
